# Intelligent Real-Time Face-Mask Detection System with Hardware Acceleration for COVID-19 Mitigation

**DOI:** 10.3390/healthcare10050873

**Published:** 2022-05-09

**Authors:** Peter Sertic, Ayman Alahmar, Thangarajah Akilan, Marko Javorac, Yash Gupta

**Affiliations:** Department of Software Engineering, Lakehead University, Thunder Bay, ON P7B 5E1, Canada; pisertic@lakeheadu.ca (P.S.); aalahmar@lakeheadu.ca (A.A.); mjavorac@lakeheadu.ca (M.J.); ygupta1@lakeheadu.ca (Y.G.)

**Keywords:** computer vision, COVID-19 mitigation, deep neural network, embedded systems, face-mask detection, hardware acceleration

## Abstract

This paper proposes and implements a dedicated hardware accelerated real-time face-mask detection system using deep learning (DL). The proposed face-mask detection model (MaskDetect) was benchmarked on three embedded platforms: Raspberry PI 4B with either Google Coral USB TPU or Intel Neural Compute Stick 2 VPU, and NVIDIA Jetson Nano. The MaskDetect was independently quantised and optimised for each hardware accelerated implementation. An ablation study was carried out on the proposed model and its quantised implementations on the embedded hardware configurations above as a comparison to other popular transfer-learning models, such as VGG16, ResNet-50V2, and InceptionV3, which are compatible with these acceleration hardware platforms. The ablation study revealed that MaskDetect achieved excellent average face-mask detection performance with accuracy above 94% across all embedded platforms except for Coral, which achieved an average accuracy of nearly 90%. With respect to detection performance (accuracy), inference speed (frames per second (FPS)), and product cost, the ablation study revealed that implementation on Jetson Nano is the best choice for real-time face-mask detection. It achieved 94.2% detection accuracy and twice greater FPS when compared to its desktop hardware counterpart.

## 1. Introduction

The world faces its greatest pandemic since the 1918 influenza pandemic, severe acute respiratory syndrome coronavirus 2 (SARS-CoV-2; COVID-19). After the first confirmed cluster in December 2019 [1], COVID-19 garnered global attention. The global economy weakened as government responses, such as lockdowns and the shutdown of businesses, put regular daily life on hold. With international travel slowdown, the Air Transport Association (ATA) projected a 2020 loss of passenger carriage revenue of up to USD 314 billion compared to 2019 (IATA Economics’ Chart of the Week: “recovery in air travel expected to lag economic activity”—https://www.iata.org (accessed on 13 February 2022)). The International Monetary Fund (IMF) reported a drop in global GDP of −3% from 6.3% in January 2020, rendering this a greater recession than both the Great Depression and the 2009 financial crisis [2]. Mental health, the economy, and global politics have all seen negative consequences as a result. The prevalence of adverse psychiatric outcomes among the general public such as anxiety and depression was greater than that before the outbreak [3]. Many nonessential workers with lower financial status are more susceptible to financial hardships due to job loss [4,5]. With these negative consequences, it is vital that the pandemic ends as quickly as possible. Mass vaccinations are taking place, and they have been effective at reducing hospitalisations and deaths. However, nonpharmaceutical interventions in the form of social distancing and mask mandates are still very important [6]. As such, countermeasures such as mask-wearing compliance must be employed to slow the spread of the virus and its new variants. It is the simplest and most popular measure for stopping the spread of the virus. However, it is only effective if most people (>70%) agree to adhere to it [7]. Those who do not adhere to the recommendations allow for the virus to spread and lengthen the pandemic’s effects. Thus, there must be a way to enforce the mask-wearing policy without requiring a tremendous amount of resources behind it. In this direction, the contributions of this research are proposing a novel automated face-mask detection solution that is cost-effective and was designed for embedded hardware with dedicated machine-earning workload accelerators. This work offers the following contributions.

1. A cost-effective technological solution is proposed for automatic face-mask detection. It exploits computer-vision (CV)-based face detection in conjunction with machine-learning strategies to build an integrated system that classifies detected faces as having a mask on or not. Such a system, which is provided as an open-source or a commercial product, can be implemented in many public and private sectors and businesses in order to ensure that the local population is adhering to mask-wearing policies.
2. Three hardware-specific quantised models were built and benchmarked for further real-time system implementations.
3. A thorough ablation study was conducted to prove the effectiveness of the proposed face-mask detection system on embedded hardware. The proposed model’s performance was also compared with that of other state-of-the-art DL approaches using evaluation metrics of accuracy, inference time, memory footprint, and cost.

The rest of this paper is organised as follows. Section 2 describes key works from the literature. Section 3 outlines important background details. Section 4 elaborates on the proposed model and its benchmarking on various hardware platforms. Section 5 analyses the experiments. Lastly, Section 6 concludes the paper with future research directions.

## 2. Literature Review

Face-mask detection is fundamentally intertwined with facial recognition due to their visual and technological overlap. The foundation laid by facial recognition technologies over the past decade can directly translate to the new implementations of face-mask detection. For example, Mercurio [8] utilises the MediaPipe framework and some of its prebuilt solutions for building models for this task. The MediaPipe framework allows for easy use and optimised machine-learning algorithms for a wide range of hardware. The authors chose the hand, face mesh, and pose solutions for the real-time processing of the input video feed. Chavda et al. [9] described the difficulties with manually tracking face-mask compliance and suggested a solution built on a multistage convolutional neural network (CNN) targeted for a specific CCTV camera. Their architecture was composed of two unique CNNs, one for face detection and one for mask classification. The authors noted that this approach allowed for them to build on high-performing pretrained face-detection models, such as Dlib [10] or RetinaFace [11]. Once a face is detected at the first stage, an intermediate process is carried out for region-of-interest (ROI) extraction along with image resizing and normalisation. Once image patches had been collected, the authors applied three preproduced classifiers to classify them as “mask” and “no mask”. A unique aspect of their approach is that their model classified cases such as improperly worn masks and hand-covering faces as “no mask” to achieve more accurate classification performance. Liu et al. [12] proposed a classifier based on a single-shot detector (SSD) architecture. While this construct suggests only one model, a more accurate description is explained as using the base model of VGG-16 for feature extraction and then truncating the model prior to classification. After this truncation, a variety of feature layers are added, and a custom model is built with MobileNetV2, ResNet50, and Xception for the final classification. Wu et al. [13] proposed a face-mask detection method to monitor whether people wear face masks in a right way in public.The feature extractor combined the Res2Net module and deep residual network to extract information from the input by utilising a hierarchical convolutional structure, deformable convolution, and nonlocal mechanisms. An enhanced path aggregation network (En-PAN) was applied for feature fusion. In addition, localisation loss was adopted in the model training phase, and the Matrix NMS method was used in the inference stage to improve detection efficiency. Song et al. [14] recommended a face-mask detection and facial-recognition system that supports mask position and type detection. The authors introduced a stacking ensemble model learning framework based on machine-learning feature extraction, deep-learning models, and transfer learning as key algorithm support for the developed system.

With the growing demand for faster and more robust DL-based systems, many resources and efforts are applied to hardware-based improvements. Such hardware-accelerated implementations are projected to reach a USD 67 billion market in the coming years (from hundreds of use cases, https://www.mckinsey.com/ (accessed on 13 February 2022)). For instance, Reuther et al. [15] surveyed this new frontier and provided a high-level overview of how different methods and classes of accelerators perform with respect to computational throughput and power consumption. The authors described how different architectures support different application areas, ranging from low-power chips designed for edge applications to neuromorphic research chips [16]. Hardware acceleration is important for COVID-19-related systems (such as face-mask detection) because it enhances the efficiency and processing speed of software systems compared to software running on a general-purpose central processing unit [17]. This helps in achieving the necessary processing speed for real-time face-mask detection results. Thus, this research explored three instances of hardware acceleration of a light DL-based face-mask detection model, and benchmarked their performance and costs. The COVID-19 research space includes studies that help in streamlining the supply chain for COVID-19-related equipment such as face masks and ventilators. For example, Riaz et al. [18] proposed algorithms to help in selecting proper ventilator manufacturers for patients with COVID-19. Compared with related research, this is the only study that provides detailed cost analysis to help the industry adopt the most cost-effective solution on the basis of its needs. Furthermore, we built three different hardware-specific quantised models that were compared for further real-time industrial implementations.

## 3. Background

This section provides a brief overview of key methodologies and strategies used in this work.

### 3.1. Deep Neural Network (DNN)

A DNN is an information-processing paradigm that was inspired by the way in which biological nervous systems such as the brain process information. DNNs are composed of a large number of highly interconnected processing elements or neurons that simultaneously work to solve a specific problem. DNNs can be trained for a variety of tasks, but are often resource-intensive, especially with larger networks [19]. A simple solution to this is to convert the model into a simpler but equivalent model that can run on optimised hardware. However, due to limited support of the model topology, pretrained models that had been confirmed to run on these optimised hardware can be tailored to a specific use-case via transfer learning and model quantisation.

### 3.2. Transfer Learning (TL)

TL is the process of taking a model that is already fully trained and adding new layers that are retrained using a custom problem dataset [20,21]. This allows for the quick training and deployment of robust models without using massive amounts of training data and resources. For a mask-detection system, DNN-based classification models such as ResNet50 or MobileNetV2, which are pretrained on a large dataset such as ImageNet, can be quickly trained on a dataset for face-mask detection problems.

### 3.3. Quantisation

A fully trained DL model such as a DNN contains a very large number of parameters and weights. The larger the network is, the more parameters it comprises. Hence, the size of a DNN poses a problem during deployment on a small system for on-device applications, for example, mask detection using Raspberry Pi. In order to fit a DL model on a low-end embedded platform and improve its inference efficiency without much compromise on accuracy, it is crucial to follow a systematic strategy to compress networks. To this end, model quantisation and knowledge distillation or transfer-learning strategies were successfully used for model optimisation [22,23]. In this work, the former was exploited. Model quantisation represents the weights of a DL model using a smaller data format by switching from 32-bit floats to 8-bit integers. This dramatically reduces memory consumption and computational costs, and also allows for compatibility between model and acceleration hardware, such as Coral Edge TPU, which operates using 8-bit integers. There are two widely used key quantisation strategies in hardware acceleration, as outlined below.

1. Dynamic range quantisation: This statically quantises parameters from floating points to integers and dynamically quantises activations during inference. At inference, weights are converted from 8-bits of precision to floating points and computed using floating-point kernels. This conversion is performed once and cached to reduce latency. To further improve latency, dynamic-range operators dynamically quantise activations on the basis of their range to 8-bits, and perform computations with 8-bit weights and activations. This optimisation provides latency close to fully fixed-point inference. However, outputs are still stored using floating-point, so that the speed-up with dynamic-range OPS is less than that of full fixed-point computation.
2. Full integer quantisation: This method statically quantises all weights and activations to INT8; therefore, it achieves the least latency during inference. In order to statically reduce precision to 8 bits, the method requires a small representative dataset. This is a generator function that provides a set of input data that is large enough to represent typical values. It allows for the converter to estimate a dynamic range for all variable data. After the representative dataset is created, the model is converted into a TFLite model (https://www.tensorflow.org/lite/convert/ (accessed on 2 February 2022)) and is hence quantised.

## 4. Proposed System

**Figure 1:**
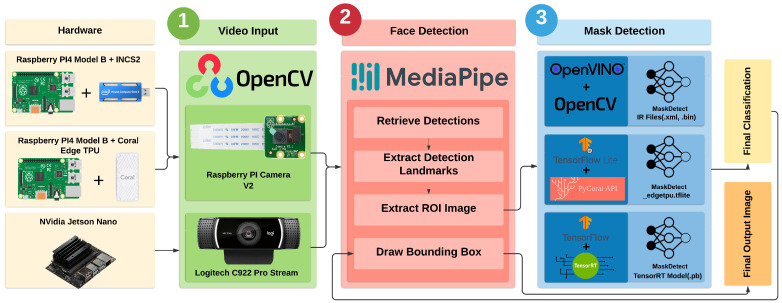
Overview of the proposed face-mask detection system that considers the combination of NVIDIA Jetson Nano 2 GB, Coral Edge TPU, Raspberry Pi4 Model B 8 GB, and INCS2 for three different hardware implementations. Video input module provides live video stream to the face-detection subsystem (FDS). FDS extracts facial regions from the input and sends them to the mask-detection subsystem (MDS). MDS uses proposed optimised hardware-specific MaskDetect to classify inputs. Classification is sent back to FDS, which then uses it to create a labeled bounding box onto the final output feed.

Figure 1 overviews the proposed hardware implementations of the face-mask detection system, which consists of the three following subsystems: video input, face detection, and mask detection. The video-input subsystem is essentially the video-input acquisition module where camera hardware is initialised, and images are captured using the OpenCV library and are sent to the second subsystem. The face-detection subsystem is responsible for face detection and ROI extraction. This subsystem is implemented using a face-detection algorithm from the MediaPipe API. The API analyses the video feed and retrieves landmark detection data that are used to calculate the ROI and extract a face image. The extracted image is then sent to the third subsystem, the mask-detection system (MDS). The MDS is responsible for classifying whether a face is wearing a mask or not. The classification is then sent back to MediaPipe where a labeled bounding box is drawn onto the input image and displayed in real time. The mask detection module, which is the proposed custom CNN model, is called MaskDetect. While running on the Raspberry Pi 4 computational platform, MaskDetect was accelerated on the hardware level using either Coral USB TPU or Intel Neural Compute Stick 2. While running on Jetson Nano, MaskDetect was accelerated using 128 Maxwell cores.

### 4.1. Proposed MaskDetect

**Figure 2:**
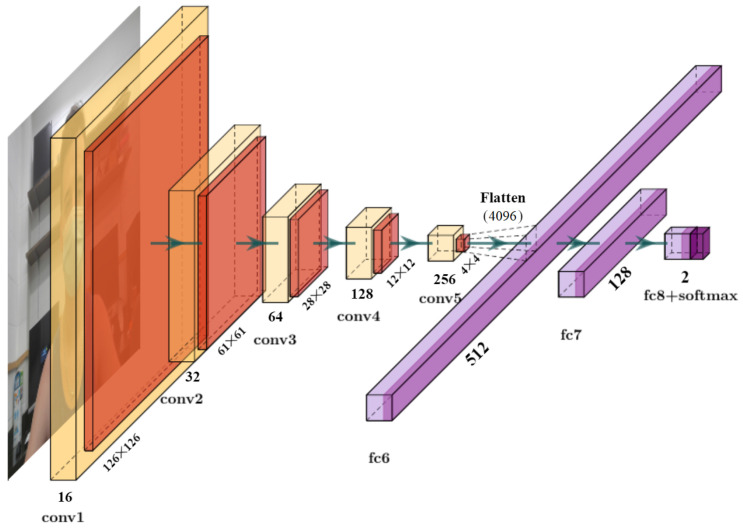
Illustration of proposed MaskDetect, which consists of five convolutional layers. The output feature map’s spatial dimensions and number of generated feature maps are shown to the side of and below each layer, respectively. Each conv. layer uses standard nonlinear activation function ReLU, followed by maxpooling operation with kernel size of 2×2. At the top, there are two fully sequentially connected layers. The final layer is two-way output with softmax.

Figure 2 depicts the proposed CNN-based face-mask classification model that is suitable for any type of spatial data such as images. In this case, it accepted an RGB video frame with dimensions of 128×128, and used the first five convolutional layers to perform feature extraction and the remaining top layers for classification, as the final layer was two-way output using a softmax activation function. Each convolutional operation uses a 3×3 kernel with no zero padding and stride of 1 followed by a maxpooling operation with a kernel size of 2×2 and stride of 2. Thus, through these repeated operations of convolution and subsampling, the final convolutional layer generates 256 output feature maps with a spatial dimension of 4×4. It is then flattened and connected to a 512-neuron dense layer. The penultimate layer is also a dense layer with 128 neurons. Convolutional and fully connected layers (except the classifier layer) use ReLU as the nonlinear activation function. Thus, the model had a total of 983,330 trainable parameters (cf. Table 1). The model was trained for 5 epochs using a categorical cross-entropy loss function and the Adam optimiser with a batch size of 32. The model’s performance was compared to that of other pretrained architectures that were compatible with the acceleration hardware used in this study, and it is discussed in Section 5.

### 4.2. Deployment on Embedded Platforms

This work aims to implement a face-mask detection system that could be deployed in any environment without needing a high-performance computing platform. Thus, we carried out a market search on cost-effective commercial off-the-shelf (COTS) embedded platforms that were suitable for this work. Through this market study and empirical analysis, we identified the following hardware platforms in which the optimised face-mask detection inference model could be deployed: Intel Neural Compute Stick 2, NVIDIA Jetson Nano, and Google Coral Edge TPU. Algorithm 1 summarises the model deployment steps tailored to the specific embedded devices above.
**Algorithm 1:** Algorithmic summary of model deployment tailored to specific embedded devices used in this work  
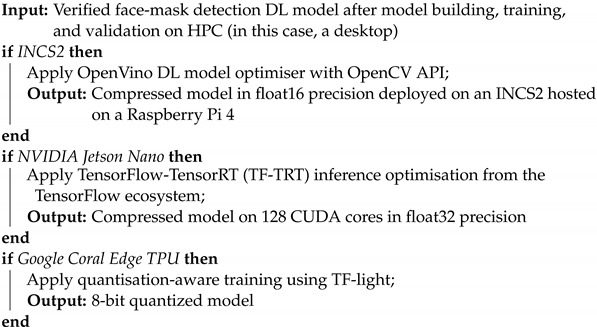


#### 4.2.1. Intel Neural Compute Stick 2 (INCS2)

This accelerator is a Movidius Myriad X vision processing unit (VPU) that is dedicated to image-based applications and inference. The 16 programmable vector microprocessors allow for concurrent vision pipelines with up to 1 TPOS of computing performance. Although the form factor of a USB stick would not be traditionally perceived as an embedded system, the idea is to provide an affordable and accessible tool to help in developing and prototyping systems before moving to large-scale manufacturing with integrated components. This lends itself to advanced mobile vision applications such as drones and, in our case, remote monitoring.

INCS2 provides accelerated inference performance on DNNs using Intel’s OpenVino toolkit. Intel provides working pretrained models through the Open Model Zoo repository, which can be converted into a compatible intermediate representation (IR) format with the OpenVino inference engine. Custom models from DL programming paradigms Tensorflow, Caffe, and PyToch can also be converted provided that all operations in the network are supported by the inference engine. Using the OpenVino model optimiser, MaskDetect was converted into a float16 precision model stored in its IR format. Using the OpenVino-OpenCV API, MaskDetect was deployed on the INCS2 hosted on Raspberry Pi 4.

#### 4.2.2. NVIDIA Jetson Nano

The Jetson Nano provides a complete hardware solution for accelerating DNN workloads for embedded applications. The Tensorflow-TensorRT API provides translation utility to accelerate face-detection and mask-classification models on its 128 CUDA cores. Using the API, MaskDetect was converted into an optimised float32 TensorRT model and executed using the TensorFlow library. This implementation was the simplest since it did not require an additional hardware accelerator, unlike Coral and INCS2. NVIDIA’s GPUs are used to accelerate these kinds of workloads, and NVIDIA provides a rich set of documentation for rapid testing.

#### 4.2.3. Coral Edge TPU

This system utilises the Google Coral Edge TPU for MaskDetect acceleration. Coral is a general-purpose machine-learning platform for edge applications. It can execute TensorFlow Lite models, which are lightweight and resource-efficient when compared to their TensorFlow counterparts. Due to its small form factor and limited resources, the coral stick requires an 8-bit quantised model for faster performance. There are two ways to run models on Coral: (1) finetuning pretrained models by using pretrained models and finetuning them depending on application; and (2) quantising custom models, where the custom model must be quantised and compiled using the Edge-TPU compiler for Coral. In this work, the latter technique was adopted. Before it could be deployed on the Coral USB, MaskDetect had to first undergo quantisation-aware training (QAT) in order to retain its performance as a full-integer quantised model. This is the process of retraining a model with quantisation-aware layers and a subset of training data in order for the model to adjust the layer’s parameters, such that performance is similar to that of the full-size float32 model. Once QAT is completed, MaskDetect is converted into a TFLite model using the TensorFlow Lite converter. The TFlite model was later compiled using the Coral Edge-TPU compiler for optimisation required for the Coral USB stick.

### 4.3. Transfer-Learning Models

In order to deploy MaskDetect, we had to ensure that all its components were compatible with TFLite Edge-TPU, OpenVino, and TensorRT conversions. This may not always be possible, so we could alternatively use pretrained models that are already compatible with the acceleration hardware and retrain them to our specific application using TL (TensorFlow models on the Edge-TPU, https://coral.ai (accessed on 2 February 2022)). Pretrained VGG16, ResNet-50V2, and InceptionV3 models were chosen due to their classification abilities and compatibility with different acceleration hardware platforms. Their original output classifier layers were removed, and a new classifier layer was added. These models were then finetuned for 2 epochs using our face-mask detection dataset. After retraining, models were ready to be optimised for the acceleration hardware platforms. In the case of the Coral Edge-TPU, due to INT8 hardware, models had to undergo quantisation-aware training or there was a risk of worsening the models’ detection performance.

## 5. Experimental Analysis

### 5.1. Datasets

**Figure 3:**
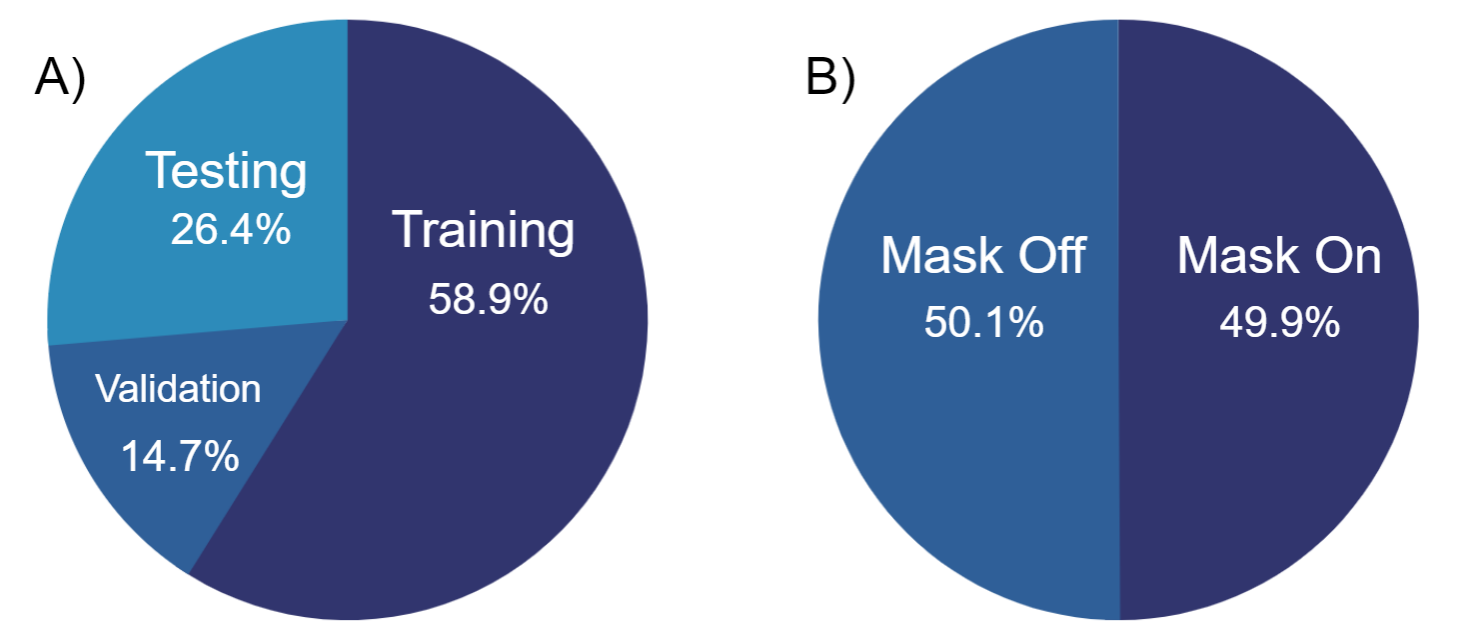
Dataset description: (**A**) data-sample and (**B**) total class distribution.

**Figure 4:**
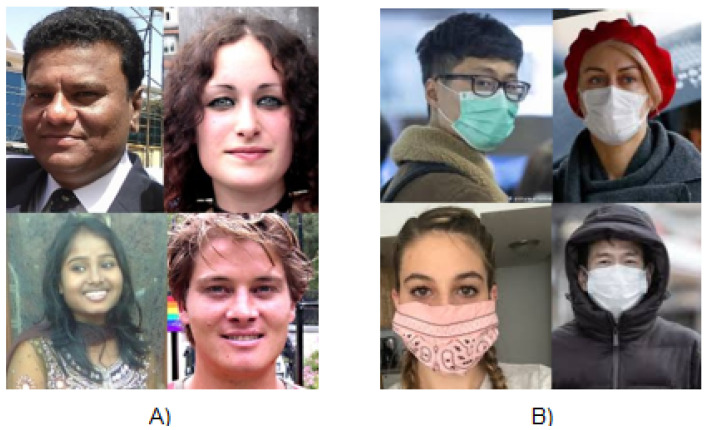
Sample images from dataset used in this study to train and validate the proposed model (**A**) without and (**B**) with mask.

The data distribution of the samples used in this study is shown in pie charts in Figure 3. A total of 3843 samples containing 1915 images of faces with masks and 1928 images of faces without masks were split with an 8:2 ratio to form training and validation sets, respectively. The testing set also comprised a mutually exclusive set of 1376 images, containing 690 samples of faces with masks and 686 samples of faces without masks. A few samples from the datasets are shown in Figure 4. Due to privacy and logistic issues relating to in-person data collection, we could not directly collect data samples from real environments, so we harvested freely licensed sample images containing the categories described above from the Google search engine. The key focus of this study is more on the embedded implementation of a face-mask detection system than actual data collection.

### 5.2. Data Preprocessing

This study exploits the following data preprocessing operations.

- Resizing: images were resized into a uniform size of 128 × 128, as the proposed MaskDetect requires uniform input dimensions;
- Normalisation: pixel intensity values were normalised to [−1, 1] for the best results from ReLu activations;
- Labeling: categorical labels were one-hot encoded.

Experimental analysis included developing and testing multiple models with different hardware accelerations. Using a high-end desktop hardware and DL models, real-time face-mask detection and classification are easily achievable. However, these models are unable to perform in real time when operating on embedded hardware, i.e., resource-limited computational platform [24]. In this study, desktop-based implementation was the baseline. The desktop consisted of an Intel I7-8650U CPU 1.9 GHz, and 16 GB RAM with a retail cost of approximately USD 409 (CPU Product Specifications—https://ark.intel.com (accessed on 14 January 2022)), which is referred to as *BaseCost*. The performance of the accelerated MaskDetect models was then compared with the baseline implementation.

### 5.3. Desktop-Based Implementations and Analysis

First, all desktop-based implementations were analysed. The MaskDetect, VGG16, ResNet-50V2, and InceptionV3 models’ performance is shown in Table 1.

The accuracy performance of MaskDetect was slightly worse than that of the retrained VGG16, ResNet-50V2, and InceptionV3, with the largest difference being 4.8% when compared to Resnet-50V2. These results show that the proposed lightweight (11.5 MB) MaskDetect model could provide comparable performance with a huge reduction in the total number of trainable parameters, resulting in faster inference speed. The model achieved an inference time of 46 ms, which was faster than that of all other transfer-learning models. This inference speed is suitable for real-time applications. This is vital for embedded hardware-based deployments, as there are often little available memory and few computational resources. Given the results, MaskDetect is an ideal choice for deployment on any dedicated embedded hardware. Thus, we extended the implementation on various platforms via model quantisation, and analyse performance in the following subsections.

### 5.4. Quantisation Analysis

Quantisation was utilised in this work in order to compress the neural network. This compression facilitated the deployment of the system for hardware acceleration. In this work, quantisation compressed the weight of the DL model by switching from 32-bit floats (FP32) to 8-bit integers (INT8). This helped the system in accomplishing two objectives. First, achieving a large reduction in computational time and memory requirements. Second, facilitating compatibility between model and acceleration hardware. Table 2 compares the performance of the optimised MaskDetect model achieved via quantisation-aware training with that of the desktop-based baseline model. Quantisation-aware training drastically reduced the size of the model from 11.5 to 0.983 MB while improving face-mask classification accuracy by 0.8%. Size in the table refers to the model’s memory occupation, and classification accuracy was obtained using a computational desktop platform with an Intel I7-8650U CPU 1.9 GHz and 16 GB RAM.

### 5.5. Extended Experiments on More Hardware Accelerations

**Figure 5:**
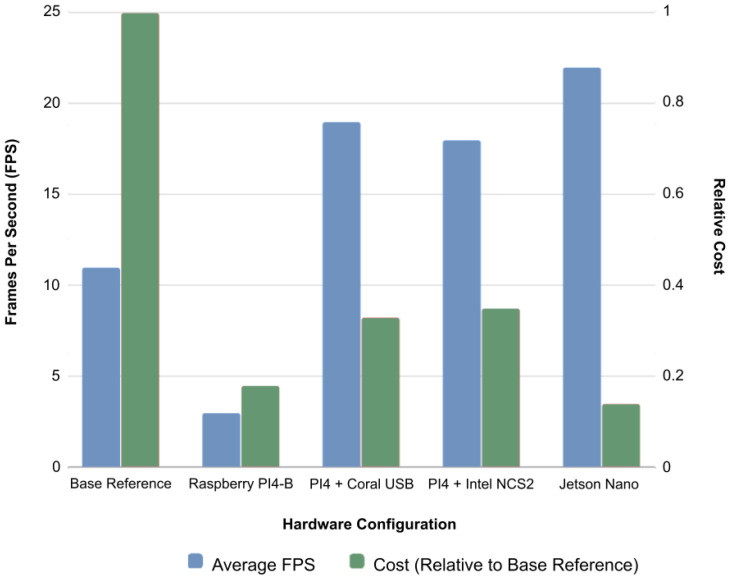
Performance of MaskDetect running on different hardware configurations. Cost of each hardware system measured relative to cost of baseline desktop. The former two hardware configurations were with desktop-based MaskDetect; the latter were their optimised variants.

**Figure 6:**
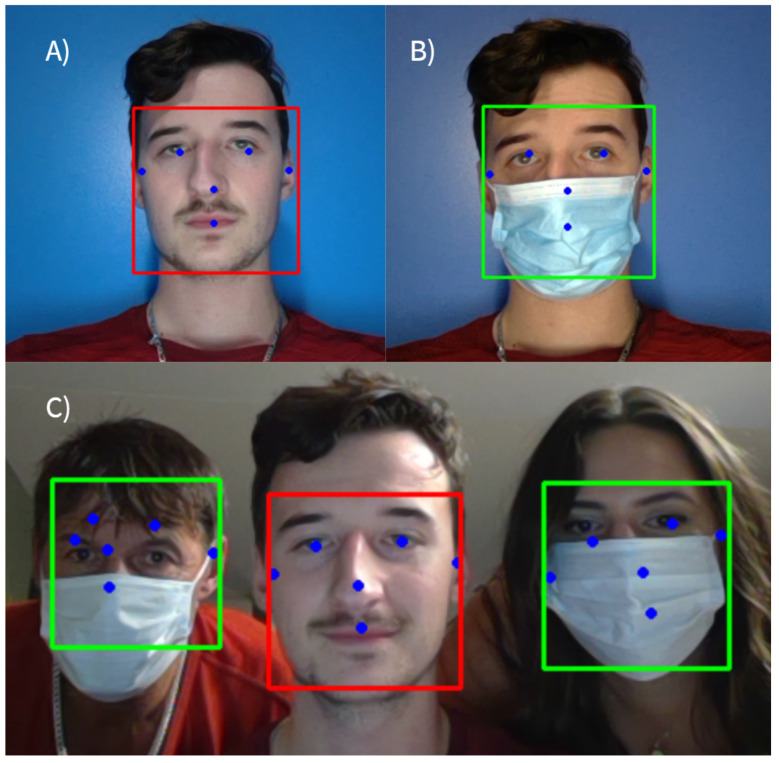
Sample outputs of baseline MaskDetect: (**A**) single-instance detection with mask off; (**B**) single instance detection with mask on; (**C**) multi-instance detection output.

This work mainly focuses on the implementation of the proposed face-mask detection model MaskDetect on embedded hardware accelerators. Thus, MaskDetect was implemented and accelerated on different embedded platforms that require different strategies for model quantisation to be compatible with the targeted hardware. Thus, variation in the quantisation pipeline and the hardware’s design methodology resulted in varying real-time performance and detection accuracy, as shown in Table 3 and Figure 5. To meet a real-time performance, this study focuses on frames-per-second (FPS) performance. Figure 6 shows the qualitative results of the proposed MaskDetect’s baseline implementation.

1. Coral USB-based acceleration: This implementation accelerated the MaskDetect model on Raspberry Pi 4 using the Coral Edge USB at a relative cost of approximately 0.33 *BaseCost* (Pi product specifications: https://www.pishop.us (accessed on 1 February 2022); Coral product specifications: https://coral.ai (accessed on 1 February 2022)). The MaskDetect-Coral configuration achieved the second fastest real time performance (cf. Table 3) among the three hardware accelerated implementations, which could be attributed to the INT8 quantisation of the model. The system achieved average performance of 19 FPS, increasing the real-time performance of MaskDetect by nearly 73%. However there was a slight reduction detection accuracy due to weight quantisation, resulting in accuracy of 90.4%.
2. INCS2-based acceleration: This implementation accelerated the MaskDetect model on Raspberry Pi 4 using INCS2 VPU at a relative cost of approximately 0.355 *BaseCost* (Pi product specifications: https://www.pishop.us, (accessed on 1 February 2022) INCS2 product specifications: https://store.intelrealsense.com (accessed on 1 February 2022)). The MaskDetect-INCS2 configuration achieved the second fastest real-time performance of 18 FPS, increasing the performance of MaskDetect by nearly 64%. Since the INCS2 model ruranns at FP16 quantisation, there was no measurable accuracy loss when compared to the original MaskDetect model.
3. Jetson Nano acceleration: This implementation accelerated the MaskDetect model on the Jetson Nano Developer Kit using the 2 GB hardware version at a relative cost of approximately 0.147 *BaseCost* (Jetson product specifications: https://www.amazon.com (accessed on 1 February 2022)). This configuration provided the best real-time performance of 22 FPS. This resulted in an increase of MaskDetect’s performance to 100%. Real-time performance was the quickest on the Jetson Nano due to its GPU hardware, which is not available on Raspberry Pi. There was no measurable accuracy loss, as the model used FP32 weights, matching the accuracy of the baseline model.

### 5.6. Cost Analysis

When comparing acceleration hardware with the desktop-based baseline (cf. Table 3), experiments demonstrated that superior performance could be achieved at a fraction of the cost. Both Coral USB and Intel NCS2 require additional hardware to function, while Jetson Nano works as a standalone system. This gives the Jetson Nano a significant technical advantage, as vertical integration allows for easier implementation. This also allows for the implementation of the face-mask detection system at under half the cost of the Coral and INCS2 implementations. NVIDIA’s documentation regarding deployment and model conversion was significantly better than that of Google and Intel’s offerings, allowing for much faster experimentations. Taking in these factors, the Jetson Nano design is recommended by this work for a consumer-grade product. However, with a streamlined system that optimises the entire software process aside from model inferencing, the INCS2 and Coral implementations could be expected to match or even outperform that of Jetson. Future works will focus on this area of improvement.

## 6. Conclusions and Future Direction

Over the past two years, wearing a face mask became the best defence against COVID-19 and other airborne viruses. This assumption is based on whether the public complies with local and federal regulation regarding their use, particularity in indoor settings. This research introduced a novel face-mask detection system designed for embedded hardware with dedicated machine-learning workload accelerators. We designed and developed a custom CNN model called MaskDetect that is capable of classifying in real time whether or not an individual within an input video stream is wearing a mask. Implementations focused on three unique hardware systems, namely, Google’s Coral USB, Intel’s NCS2, and NVIDIA’s Jetson Nano. The performance and costs–benefits of these implementations were thoroughly analysed. The NVIDIA Jetson Nano was the simplest and lowest-cost solution in the design and implementation challenges while also providing the fastest real-time performance. A limitation of this work is that it considered only three hardware platforms. Therefore, future research should experiment with more platforms to expand the space of hardware acceleration. Future work could also add risk priority number assessment. This preliminary research enables public and private industries and institutions to deploy automated mechanisms to ensure face-mask compliance, and advance the field of machine-learning accelerators and intelligent embedded computer-vision solutions.

## Figures and Tables

**Table 1:** Comparative performance analysis of various models on a desktop computational platform with Intel I7-8650U CPU 1.9 GHz and 16 GB RAM.

Model Name	Classification Accuracy	Model Size in Memory (MB)	Number of Parameters	Inference Time (s)
MaskDetect (baseline)	0.942	11.5	983,330	0.046
VGG16	0.987	43.8	4,945,858	0.049
ResNet-50V2	0.990	143.0	27,825,583	0.050
ResNet-50V2	0.974	103.0	22,917,794	0.055

Note: implementations of VGG16, ResNet-50V2, and ResNet-50V2 are based on pretrained ImageNet models available on open-source Keras libraries-Module:tf.keras.applications—https://tensorflow.google.cn/api_docs/python/tf/keras/applications/ (accessed on 10 February 2022).

**Table 2:** Impact of quantisation-aware training.

Model Name	Size (MB)	Classification Accuracy
MaskDetect (baseline)	11.5	94.2%
QAT MaskDetect	0.983	95.0%

**Table 3:** Performance analysis of various implementations of MaskDetect regarding computational platforms.

Hardware	Avg. FPS	Accuracy	Model	Relative Cost	Extra Hardware
Baseline	11	0.942	MaskDetect	*BaseCost*	No
Raspberry P14-B	3	0.942	MaskDetect	0.183 × *BaseCost*	No
P14+Coral USB	19	0.904	MaskDetect_edgeTPU.tflite	0.330 × *BaseCost*	Yes
P14+Intel NCS2	18	0.943	MaskDetect_IR	0.355 × *BaseCost*	Yes
Jetson Nano	22	0.942	MaskDetect_TensorRT	0.147 × *BaseCost*	No

## Data Availability

Not applicable.

## Abbreviations

The following abbreviations are used in this manuscript:

CV	Computer vision
CNN	Convolutional neural network
DL	Deep learning
DNN	Deep neural network
FPS	Frames per second
FDS	Face-detection subsystem
INCS2	Intel Neural Compute Stick 2
IR	Intermediate representation
MDS	Mask-detection system/subsystem
QAT	Quantisation-aware training
ROI	Region of interest
SSD	Single-shot detector
TL	Transfer learning
VPU	Vision processing unit
